# Age effect explorer: a Shiny application to browse and visualize tissue-specific age-related gene expression changes

**DOI:** 10.1093/bioadv/vbag026

**Published:** 2026-01-29

**Authors:** Menghui Chen, Mingrui Li, Ronnie Y Li, Jie Jiang, Zhaohui S Qin

**Affiliations:** Department of Biostatistics and Bioinformatics, Emory University, 1518 Clifton RD NE, Atlanta, GA 30322, United States; Department of Biostatistics and Bioinformatics, Emory University, 1518 Clifton RD NE, Atlanta, GA 30322, United States; Stravitz-Sanyal Institute for Liver Disease and Metabolic Health, Virginia Commonwealth University, 1200 East Broad Street, West Hospital, Richmond, VA 23298, United States; Department of Cell Biology, Emory University, 615 Michael Street, Atlanta, GA 30322, United States; Department of Biostatistics and Bioinformatics, Emory University, 1518 Clifton RD NE, Atlanta, GA 30322, United States

## Abstract

**Motivation:**

Understanding age-related transcriptional changes in human tissues is crucial for elucidating molecular mechanisms of aging and disease. Current genomic analysis tools often require programming expertise, limiting accessibility for comprehensive aging studies. Here, we present Age Effect Explorer, an interactive R Shiny application for systematically analyzing age- and sex-related gene expression pattern changes across 54 human tissues using Genotype-Tissue Expression (GTEx) v10 data.

**Results:**

We obtained gene-level expression profiles from 981 individuals, and fitted ordinary least squares linear models including age, sex, and technical covariates with FDR correction. Pre-calculated results are stored in a cloud database enabling rapid, code-free exploration through an intuitive web interface. Age Effect Explorer validated known aging markers including age-correlated EDA2R. This resource democratizes access to aging transcriptomics, facilitating the discovery of tissue-specific aging mechanisms.

**Availability and implementation:**

The Age Effect Explorer can be accessed using a web browser at https://menghui.shinyapps.io/ageeffectexplorer/. The code used to create the Shiny application, along with a tutorial, can be found on GitHub at https://github.com/ML198/GTEx-Explorer.

## 1 Introduction

Gene expression regulation plays a critical role in maintaining cellular and tissue function in the human body. Expression levels of many genes vary not only between individuals but also across different tissues and development stages within the same individual. Among the various factors influencing gene expression, age has been consistently shown to have a significant impact across diverse organs and tissues (Glass *et al.* 2013, Harris *et al.* 2017, Jia *et al.* 2018, Viñuela *et al.* 2018, Yamamoto *et al.* 2022).

Understanding age-associated changes in gene expression is paramount for comprehending the molecular mechanisms underlying the human aging process and age-associated disorders. It is also of great interest to systematically identify age-related genes to uncover novel biological insights from them. Previous studies have demonstrated that aging is characterized by widespread transcriptional alterations, including dysregulation of immune response genes, mitochondrial dysfunction pathways, and DNA repair mechanisms (Martinez-Jimenez *et al.* 2017, Srivastava 2017, Santoro *et al*. 2021, Sims and Gurkar 2023, Somasundaram *et al.* 2024). However, most aging research has focused on individual tissues or limited gene sets, lacking the comprehensive perspective necessary to understand systemic aging processes. Recently, the Genotype-Tissue Expression (GTEx) project (GTEx Consortium 2017) provided complete gene expression profiles for many somatic tissues sampled from a large cohort of donors across a wide age range, offering an unprecedented opportunity to investigate age-related transcriptional changes in the human body.

Despite the wealth of data available through GTEx, genomic datasets often present barriers to access, analyze and visualize, particularly for investigators without extensive bioinformatics training. Current tools for exploring GTEx data typically require programming skills or provide limited functionality for age-specific analyses (Stanfill and Cao 2021, Guzzi *et al*. 2023). This gap highlights the need for user-friendly, interactive platforms that can democratize access to these valuable genomic resources while maintaining analytical rigor.

In this study, utilizing the unique GTEx resource, we conducted a comprehensive analysis of age-related gene expression changes by applying linear regression models to quantify the effect of age on gene expression across multiple somatic tissues, adjusting for multiple known covariates and confounding factors. To make these findings broadly accessible to the research community, we developed Age Effect Explorer, an interactive R Shiny application that enables users to visualize age-related expression patterns for genes and tissues of interest. To illustrate its utility, we present examples of genes showing significant age-related expression changes in somatic tissues. We believe Age Effect Explorer is an intuitive and effective tool for researchers to explore the gene expression dynamics in human development and aging.

## 2 Methods

### 2.1 GTEx data

**Figure 1:**
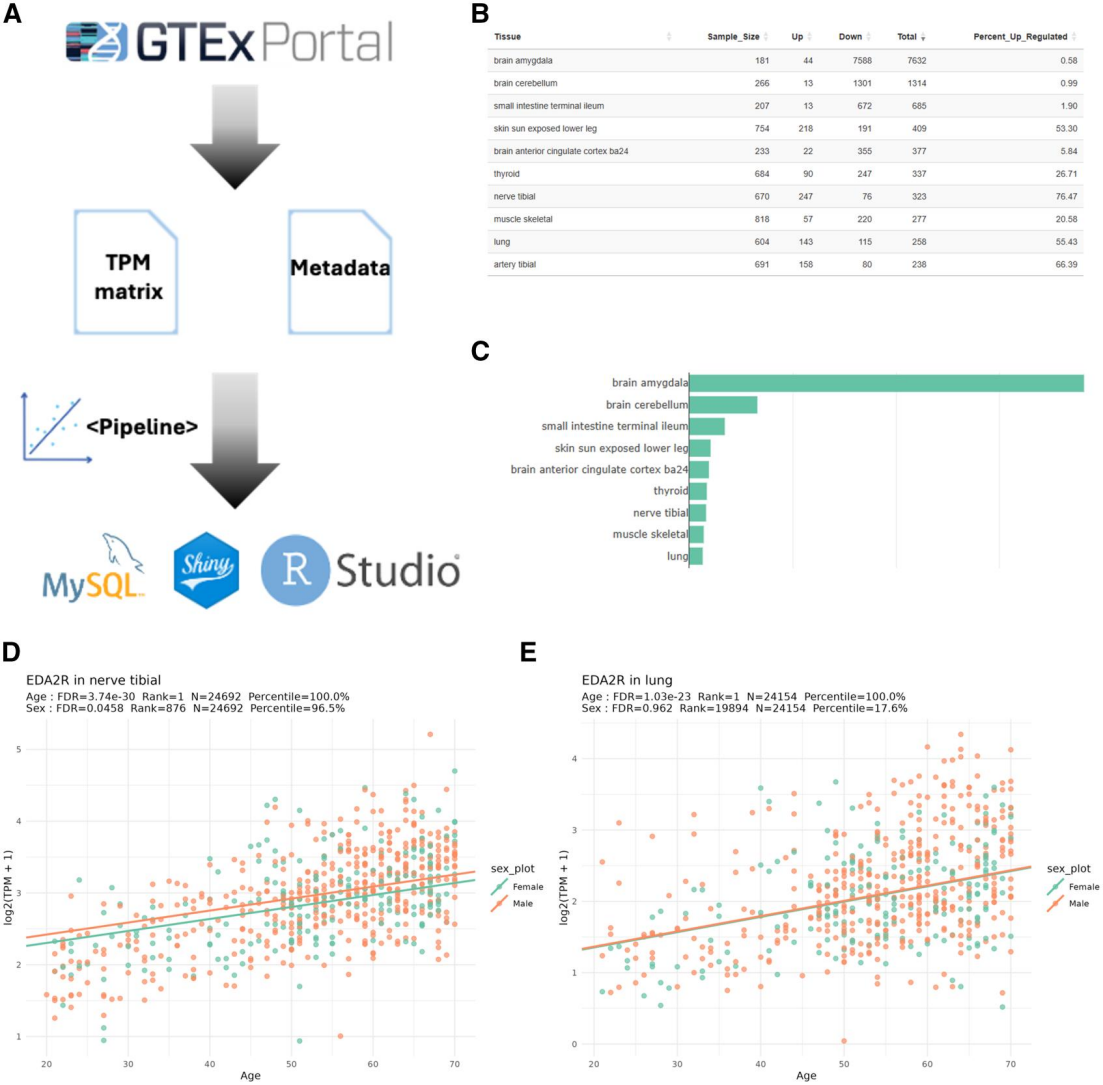
Schematic of Shiny application development and features. (A) Data processing pipeline overview. TPM matrices and metadata were downloaded from the GTEx Consortium (version 10), subjected to a processing pipeline, and deployed in a Shiny application, making the dashboard accessible on a web browser. (B and C) Example screenshots of the dashboard. Features include browsing age-related genes collectively or individually. This visual depicts the percentage of upregulated genes in a subset of GTEx tissues. (D) Expression of ectodysplasin A2 receptor (EDA2R) gene in nerve tibial shows a positive association with age (*P*-adj = 3.74 × 10^–30^). The sex effect was significant and showed that males had a higher baseline expression than females (*P*-adj = 0.0458). (E) Expression of EDA2R gene in the lung also displays a significant age-related increase (*P*-adj = 1.03 × 10^–23^). Expression differences between sexes in this tissue were not statistically significant.

We obtained publicly available, multi-tissue gene-level expression data (in transcripts per million, TPM) from version 10 of the GTEx portal, encompassing 54 non-diseased human tissue types from a total of 981 donors. The number of donors per tissue varied substantially, ranging from 13 in the kidney medulla to 820 in skeletal muscle. TPM values were calculated per sample and were not normalized across samples or tissues. Donor metadata, including age and sex, were also retrieved from the GTEx portal. To account for potential confounding factors in our regression analyses, we used covariate matrices from the GTEx consortium, which included genotyping principal components (PCs), Probabilistic Estimation of Expression Residuals (PEER) factors, and indicators for sequencing protocol and platform. We incorporated the same number of PEER factors as the GTEx Consortium, which ranged from 15 factors to 60 factors based on tissue sample size. We also included the first five genotyping PCs for each tissue. Across all 981 GTEx donors, the age ranged from 20 to 70 years (median = 55 years, IQR = 16 years). The distribution of ages was skewed toward older adults (>50 years) for both males and females. Figure 1, available as supplementary data at *Bioinformatics Advances* online illustrates the general distribution of GTEx donors by sex, as well as tissue-specific age distributions.

### 2.2 Statistical modeling

For each tissue, we first downloaded the raw TPM matrices along with corresponding sample metadata (age, sex, and technical/biological covariates). Next, a log_2_(TPM + 1) transformation was applied to the TPM matrix, and a gene‐wise ordinary least squares regression model was subsequently fitted for each gene:


(1) $$y_{i,j}\;=\;\beta_{0,i}\;+\;\beta_{\text{age},i}{\mathrm{Age}}_{j}+\;\beta_{\text{sex},i}{\mathrm{Sex}}_{j}\;+\sum_{k}\gamma_{k,i}C_{k,j}\;+\;\varepsilon_{\mathrm{ij}}$$

Here, *y_i,j_* is the log-transformed TPM for gene *i* in donor *j*, *Age_j_* is a continuous variable representing age of donor *j*, *Sex_j_* ∈{0,1} is a binary indicator variable (1 for male and 0 for female donors), and *C_k,j_* denotes additional covariates. Genes with negligible variance or with more than 90% zero value across all samples within the tissue were excluded. This resulted in tissue-specific gene sets ranging from 18 061 genes (whole blood) to 34 979 genes (testis). The median number of genes tested was 23 448 genes. The Benjamini–Hochberg procedure was used on the age and sex coefficients to control the false discovery rate (FDR) (Benjamini and Hochberg 1995). Associations with FDR-adjusted *P*-value (*P*-adj) < .05 were considered statistically significant.

We chose to utilize a linear model for three main reasons. First, the linear age coefficient provides a directly interpretable effect size that can be meaningfully compared across genes and tissues. Second, our primary question concerns whether genes are consistently up- or downregulated in a tissue, so the direction and magnitude of change are more relevant than the precise functional form. Linear models effectively capture monotonic trends such as the ones we are interested in, given limited sample sizes. Lastly, linear modeling of age effects is standard practice in transcriptomic analyses, including those done by the GTEx Consortium (2017). Thus, our model facilitates comparisons with existing literature.

### 2.3 Sensitivity analyses

To assess the relationship between tissue sample size and the number of differentially expressed genes (DEGs), we performed a cross-tissue correlation analysis across all 54 GTEx tissues. We utilized Kendall’s tau, a non-parametric rank-based correlation coefficient, to quantify the association between sample size and the number of age-associated and sex-associated genes at FDR < 0.05. Additionally, we conducted a power analysis to evaluate statistical power across tissues with varying sample sizes. Power was estimated using the pwr.f2.test() function in R (pwr package), assuming a linear regression framework with one predictor of interest (age or sex) and a significance level of α = 0.05. Following Cohen’s standard conventions for multiple linear regression, three standardized effect sizes were evaluated: small (*f*^2^ = 0.02), medium (*f*^2^ = 0.15), and large (*f*^2^ = 0.35). For each tissue, power was calculated based on its sample size and the specified effect size thresholds.

To validate that observed age-associated genes represent genuine biological signals rather than false positives, we performed a permutation test across 50 tissues. For each tissue, we randomly shuffled the age labels across all samples, thereby breaking the true age-expression relationship while preserving the expression data structure, covariate relationships, and multiple testing framework. We then re-ran the identical linear regression analysis with permuted ages and applied Benjamini-Hochberg FDR correction to count the number of genes reaching significance at FDR < 0.05. This procedure was repeated 3 times for each tissue, and the mean number of significant genes under the null hypothesis was calculated. This permutation approach provides an empirical assessment of the false positive rate under our analytical framework and sample size distribution.

### 2.4 Shiny application

To facilitate visualization and exploration of age- and sex-associated gene expression patterns, we developed Age Effect Explorer, a web-based R Shiny application. The backend was implemented using a modular R codebase that integrated multiple packages for visualization (ggplot2, plotly, and DT) and data manipulation (dplyr, data.table, and tidyr). TPM expression matrices and donor metadata were stored in a secure MySQL database to enable fast query-based retrieval. To ensure both responsive rendering and reproducibility, all regression results and summary statistics were precomputed and stored as flat .csv files.

### 2.5 Gene set enrichment analysis

Gene set enrichment analysis (GSEA) (Subramanian *et al.* 2005) is a rank-based method that calculates the enrichment of groups of biologically related genes in a dataset. We implemented GSEA using the fgsea package in R (Korotkevich et al. 2021). Genes were ranked using the adjusted *P*-values of the age effect. We used the MSigDB WikiPathways database as reference gene sets (Agrawal *et al.* 2024).

## 3 Results

### 3.1 Age effect explorer interactively visualizes age and sex effects

The Age Effect Explorer interface includes two fully interactive modules, Tissue-level Summary and Age-effect browser, which together enable intuitive, code-free exploration of gene expression trend associated with aging. Figure 1 illustrates the basic architecture of the data engineering pipeline and the user interface.

The Tissue-level Summary module offers a panoramic view of differential expressions across all 54 GTEx tissues. After selecting Age or Sex at the top, a sortable table displays the sample size per tissue, the count of significantly upregulated and downregulated genes (FDR < 0.05), and the percentage of upregulated genes. The data table can be exported as a CSV file. A corresponding bar chart ranks tissues by the total number of significant genes, includes hover-over tooltips, and supports one-click export as a PNG file, giving researchers a fast and high-level overview.

The Age-effect browser module enables exploration of individual genes. Users can select a tissue and rank genes by their age or sex effect. Upon selecting a gene, three plots are generated: (i) a scatter plot of log_2_(TPM + 1) versus age with sex-specific regression lines, (ii) an age-group boxplot stratified by sex, and (iii) a residual plot with the optional sex adjustment. These plots can be exported in PNG, PDF, SVG, and JPG formats. A cross-tissue table summarizes the gene’s age and sex effect, which helps users quickly compare its effect across tissues.

### 3.2 Age-associated genes show tissue-specific distributions

Across all GTEx tissues examined, we identified 10 335 unique genes (17.5% of all 59 033 genes tested) whose expression was significantly associated with age (FDR < 0.05) in at least one tissue; among these age-associated genes, those with at least two significant tissues (*n* = 2295) included 381 (16.60%; 95% CI, 15.14–18.18%) that exhibited opposite age effects across tissues. We refer to such genes as aging genes. All the aging genes organized by tissue can be found in Table 1, available as supplementary data at *Bioinformatics Advances* online. The distributions of these aging genes are drastically different among tissues. The amygdala of the brain showed the largest number of aging genes (7632), followed by the cerebellum of the brain (1314) and the small intestine (terminal ileum) (685). Among the 54 tissues analyzed, no aging gene was identified in eight different tissues using our method: brain-hypothalamus, EBV-transformed lymphocytes, ectocervix, endocervix, fallopian tube, kidney-cortex, kidney-medulla, and minor salivary gland. A detailed summary of aging genes by tissue is provided in Table 1, available as supplementary data at *Bioinformatics Advances* online.

At the tissue level, among all aging genes, the median proportion of upregulated aging genes is 53.3%, suggesting that during the aging process, upregulated genes outnumber downregulated ones for most tissues. Interestingly, we found most central nervous system tissues (e.g. brain-amygdala, brain-cerebellum, and small intestine-terminal ileum) showed a predominance of downregulated genes in aging, usually exceeding 90%. In contrast, several peripheral tissues (e.g. heart-atrial appendage, prostate, bladder, and adrenal gland) demonstrated 100% of aging genes being upregulated. This brain-peripheral dichotomy suggests fundamentally different transcriptional responses to aging. The predominant downregulation in brain tissues is consistent with established findings of age-related decline in synaptic and neuronal gene expression, proteostasis collapse, and reduced translation capacity (Loerch *et al.* 2008, Glass *et al.* 2013, Dönertaş *et al.* 2017). In contrast, the upregulation observed in peripheral tissues may reflect compensatory or adaptive stress response programs, such as cardiac remodeling (Tracy *et al*. 2020) and tissue-specific homeostatic mechanisms that attempt to maintain organ function despite aging-related challenges (Yang *et al.* 2015).

Browsing the results, we noticed several “frequent flyer” aging genes, meaning they showed a strong age effect across many tissues. Among them, EDA2R (Ectodysplasin A2 receptor) is a gene previously reported to correlate positively with age in the whole blood (Barbera *et al.* 2025). Using Age Effect Explorer, EDA2R is identified as an aging gene in 31 tissues. The most significant age effect occurred in nerve tibial (*P*-adj = 3.74 × 10^−30^), followed by lung (*P*-adj = 1.03 × 10^−23^), and artery tibial (*P*-adj = 3.74 × 10^−17^) (Fig. 1).

Another gene is ZNF518B, or Zinc Finger 518B. This gene is predicted to enable DNA-binding transcription factor activity and is RNA polymerase II-specific. ZNF518B is identified as an aging gene in 22 tissues. The most significant age effect was found in thyroid (*P*-adj = 1.25 × 10^−24^), followed by cells cultured fibroblasts (*P*-adj = 3.80 × 10^−20^) and lung (*P*-adj = 1.36 × 10^−19^).

Lastly, PTCHD4, or Patched Domain Containing 4, is a gene predicted to be active in the cell membrane. PTCHD4 is identified as an aging gene in 23 tissues. The most significant age effect was found in esophagus muscularis (*P*-adj = 4.70 × 10^−22^), followed by colon sigmoid (*P*-adj =1.00 × 10^−17^), and artery tibial (*P*-adj = 2.20 × 10^−14^).

### 3.3 Gene set enrichment analysis reveals patterns in ribosomal protein expression

To better understand what biological functions are likely impacted by aging genes, we performed GSEA (Subramanian *et al.* 2005) on genes ranked by age effect for tissues with sample size larger than 50. From the results, we found 877 significant (*P*-adj < 0.05) pathway/tissue combinations containing 372 unique pathways (Table 2, available as supplementary data at *Bioinformatics Advances* online). The most frequently enriched pathway was “cytoplasmic ribosomal proteins,” significant in 17 different tissues. Ranking second was “mRNA processing,” significant in 15 different tissues. The pathway “Alzheimer’s disease” ranked sixth (tied) and was significant in 8 different tissues. It seems that many of these frequently appearing pathways revolve around housekeeping and other important routine biological functions. The change in the expression levels of the genes within these pathways is closely related to the aging process in the human body.

For the cytoplasmic ribosomal protein gene set, our GSEA analysis revealed an overall predominantly downregulated pattern, with 14 tissues showing significant negative enrichment scores. This finding is consistent with the established decline in protein synthesis capacity that characterizes cellular aging across most organ systems (López-Otín *et al.* 2013). However, we found that three tissues, namely esophagus muscularis, sun-exposed skin from the lower leg, and pancreas, exhibited the opposite pattern, showing striking upregulation of cytoplasmic ribosomal proteins with aging (Fig. 2, available as supplementary data at *Bioinformatics Advances* online). These tissue-specific exceptions to the general aging signature suggest that organs experiencing chronic mechanical stress, UV damage, or high metabolic demands may maintain or enhance their translational machinery as an adaptive response to preserve essential cellular functions in the face of advancing age.

### 3.4 Genes exhibit opposite age effects

Among genes with at least two significant tissues (*n* = 2295), 381 genes (16.60%) exhibited opposite age effects in different tissues. Overall, most genes are dominated by a single direction, with only a few tissues showing opposite directions (Fig. 3, available as supplementary data at *Bioinformatics Advances* online). For instance, LMO3, PCDH9, PCDH10, SLC6A15, LHFPL4, and PHLDA3 are mainly upregulated with age, while APOE and EPHA5 are mainly downregulated.

### 3.5 Genes show sex-biased expression

Regarding sex-specific genes, we examined sex-associated differential expression across all 54 tissues using the same linear regression framework. Genes with adjusted *P*-values < .05 for the sex coefficient were considered significant. Tissues showing the largest numbers of sex-associated genes included skin (both sun-exposed lower leg and non-sun-exposed suprapubic), muscle skeletal, nerve tibial, artery tibial, whole blood, and cultured fibroblasts, each exhibiting several hundred to over one thousand significant genes. LINC01597, a long non-protein coding RNA on chromosome 20, exhibited significant sex-associated expression in 50 of 54 tissues, making it one of the most consistently sex-biased genes across the GTEx dataset. These results suggest that aging primarily influences neural and vascular tissues, whereas sex effects are most pronounced in peripheral and connective tissues such as skin and fibroblasts.

Across the GTEx v10 dataset, eight tissues displayed more than 10 genes showing overlapping associations with both age and sex, including skin (sun-exposed lower leg, *n* = 51; non-sun-exposed suprapubic, *n* = 10), nerve tibial (*n* = 32), muscle skeletal (*n* = 28), lung (*n* = 17), thyroid (*n* = 16), artery tibial (*n* = 15), and mammary tissue (*n* = 10). Notably, CECR7 was recurrently identified in six tissues, pointing to a potentially shared regulatory mechanism influenced by both sex and age. Together, these analyses reveal distinct, tissue-specific transcriptional signatures underlying age and sex-related regulation across the human body.

### 3.6 The amygdala exhibits many age-associated DEGs

Among all 54 tissues analyzed, the brain amygdala displayed a remarkably high number of age-associated DEGs, with 7632 genes (32.97% of 23 149 tested genes) reaching significance at *P*-adj < 0.05. To determine whether this reflected genuine tissue-specific biology or technical artifacts, we conducted systematic sensitivity analyses. The substantia nigra (*n* = 183) had a nearly identical sample size to the amygdala (*n* = 181), yet it exhibited only 26 DEGs (0.113%), nearly 300-fold fewer, demonstrating that sample size alone does not drive this difference. Model specifications were consistent across tissues following GTEx standards, with the amygdala using 30 PEER factors, identical to other similarly sized brain regions including the substantia nigra. Additionally, we found no significant correlation between the number of tested genes after filtering and the number of significant DEGs (Kendall’s tau = −0.114, *P* = .229), suggesting that the amygdala result is not due to multiple testing scope. However, the total number of aging DEGs did correlate positively with the tissue sample size (Kendall’s tau = 0.493, *P* = 2.03 × 10^–7^), reflecting increased statistical power to detect subtler age-related effects (Fig. 3, available as supplementary data at *Bioinformatics Advances* online). Still, substantial variation in DEG counts was observed even among tissues with similar sample sizes, suggesting that factors beyond statistical power contribute to tissue-specific aging signatures.

GSEA revealed that downregulated genes in the amygdala were significantly enriched for Alzheimer’s disease-related pathways (Fig. 4, available as supplementary data at *Bioinformatics Advances* online), consistent with neuropathological evidence that the amygdala is an early site of tau pathology and exhibits prominent atrophy in early-stage Alzheimer’s disease. In contrast, other brain tissues with similar sample sizes or large numbers of DEGs, such as the substantia nigra and cerebellum, did not demonstrate the same enrichment for Alzheimer’s disease pathways in our GSEA analysis (Fig. 4, available as supplementary data at *Bioinformatics Advances* online). Collectively, these analyses indicate that the amygdala’s elevated age-associated DEG count reflects genuine heightened molecular vulnerability to aging rather than technical confounding.

### 3.7 Sensitivity analyses substantiate age-associated DEGs

Power analysis demonstrated that statistical power increases steeply with sample size, particularly for medium (f^2^ = 0.15) and large (f^2^ = 0.35) effect sizes (Fig. 5, available as supplementary data at *Bioinformatics Advances* online). For both medium and large effects, power approached nearly 100% at sample sizes around 100. Since 49 of 54 tissues (90.7%) had sample sizes exceeding 100, most tissues possessed comparable power to detect moderate-to-large age-related expression changes. However, for small effect sizes (f^2^ = 0.02), power remained substantially lower even in well-powered tissues, with larger sample sizes conferring advantages in detecting subtle expression changes. These findings indicate that while increased DEG discovery with larger sample sizes appropriately reflects greater statistical power to detect true effects, power differences alone cannot explain the dramatic variation in DEG counts across similarly sized tissues.

To empirically test whether our observed DEG counts could arise from false positives scaling with sample size, we performed a permutation analysis across 50 tissues with 3 permutations per tissue. Under the null hypothesis (no true age effects), permutation of age labels disrupts genuine age-expression relationships while preserving the data structure and statistical testing framework. Across all 50 tissues and 3 permutations per tissue, we observed extremely few false positive DEGs at FDR < 0.05, with a maximum mean of 3 DEGs in stomach tissue (Fig. 5, available as supplementary data at *Bioinformatics Advances* online). This result (orders of magnitude lower than our observed DEG counts in all tissues) demonstrates that our FDR correction effectively controls false positive rates and that the large numbers of age-associated genes we identify, particularly in tissues like the amygdala, reflect genuine biological signals rather than inflated false discovery due to sample size-dependent statistical power.

## 4 Discussion

In this study, we developed Age Effect Explorer, an interactive R Shiny application that leverages GTEx v10 data to systematically analyze age-related gene expression changes across 54 human tissues using linear regression models. Compared to existing methods, Age Effect Explorer utilized the latest and most comprehensive multi-tissue expression dataset and detected the age effect using a rigorous statistical approach. Our findings align with previous multi-tissue analyses showing tissue-specific transcriptional aging signatures. Earlier work identified synchronized age-related expression changes across tissues, with essential tissues like heart and lung showing stronger cross-tissue synchronization (Yang *et al.* 2015). Recent studies have shown that while the average heritability of gene expression is consistent across tissues, the contribution of age varies substantially (Yamamoto *et al.* 2022), consistent with our observation of dramatic differences in aging gene expression levels across tissues.

Our identification of EDA2R as an aging gene across 31 tissues validates recent groundbreaking findings. Barbera and colleagues identified EDA2R as the top-ranking gene associated with aging in a tissue-independent manner, demonstrating that strengthening of the EDA2R/EDA-A2 signaling axis triggers parainflammatory responses and suggesting this receptor as a promising pharmacological target for mitigating aging-associated phenotypes (Barbera *et al.* 2025). Our finding that EDA2R shows particularly strong age effects in nerve tibial, lung, and artery tibial may guide targeted interventional strategies.

Our GSEA analysis revealed cytoplasmic ribosomal proteins as the most frequently enriched pathway, predominantly showing downregulation with aging. Recent work demonstrated that aging exacerbates ribosome pausing, leading to increased ribosome collisions that overwhelm quality control pathways and contribute to proteostasis impairment (Stein *et al.* 2022). Multifaceted deregulation of translation machinery components at the translational level has been documented across the lifespan (Anisimova *et al.* 2020), supporting our findings. The striking upregulation of ribosomal proteins we observed in esophagus muscularis, sun-exposed skin, and pancreas suggests tissue-specific adaptive responses in organs experiencing chronic stress, UV damage, or high metabolic demands.

The amygdala exhibited 7632 age-associated genes, far exceeding other brain regions. This is strongly supported by neuropathological evidence. The amygdala has been identified as an early site for neurofibrillary tau tangle pathology in Alzheimer’s disease (Stouffer *et al.* 2024), and amygdala atrophy in early Alzheimer’s disease is comparable to hippocampal atrophy and strongly predicts cognitive function and neuropsychiatric symptoms (Poulin *et al.* 2011). Imaging studies tracking lifespan brain changes reveal that the Alzheimer’s disease model diverges from normal aging in the hippocampus before age 40, followed by the amygdala around age 40, with similar abnormality trajectories for both structure (Coupé *et al.* 2019). Pathological studies indicate that the amygdala may represent a preferential locus for the transition from relatively benign aging to more aggressive disease, with multiple protein species becoming misfolded, potentially serving as an “incubator” for neurodegenerative pathology (Nelson *et al.* 2018). Our enrichment analysis revealing Alzheimer’s disease pathways among downregulated amygdala genes provides transcriptomic evidence supporting these observations.

Age Effect Explorer offers key advantages by providing an intuitive, code-free interface that broadens access to genomic analyses for researchers without extensive computational expertise. Nonetheless, several limitations should be considered, including the non-uniform age distribution within the GTEx cohort, use of bulk tissue data that masks cell-type-specific changes, postmortem sample collection that may not represent healthy aging, predominance of European ancestry limiting generalizability, cross-sectional design that cannot distinguish aging from cohort effects, and linear modeling that may overlook nonlinear trajectories. Moreover, the differences in sample size across tissues can contribute potential bias to age effect gene detection, although statistical power is unlikely to fully explain this difference.

In summary, Age Effect Explorer is a broadly accessible resource for investigating age-related transcriptional changes across human tissues. We anticipate updating this resource for users as new releases of GTEx data become available. Given that the GTEx project continues to expand with developmental GTEx (dGTEx) and non-human primate datasets, future updates could incorporate these additional data types to enable comparative analyses across developmental stages and species. By combining systematic statistical analyses of GTEx data with interactive visualizations, Age Effect Explorer facilitates the discovery of tissue-specific aging signatures and advances our understanding of the molecular mechanisms underlying human aging.

## Supplementary Material

vbag026_Supplementary_Data

## Data Availability

Age Effect Explorer can be accessed using a web browser at https://menghui.shinyapps.io/ageeffectexplorer/. The TPM expression data across all tissues is publicly available on the GTEx Consortium website: https://gtexportal.org. The exact ages of the GTEx donors can be obtained from dbGaP under accession number phs000424.

## References

[vbag026-B1] Agrawal A , Balcı H, Hanspers K et al WikiPathways 2024: next generation pathway database. Nucleic Acids Res 2024;52:D679–89. 10.1093/nar/gkad960 37941138 PMC10767877

[vbag026-B2] Anisimova AS , Meerson MB, Gerashchenko MV et al Multifaceted deregulation of gene expression and protein synthesis with age. Proc Natl Acad Sci USA 2020;117:15581–90. 10.1073/pnas.2001788117 32576685 PMC7354943

[vbag026-B3] Barbera MC , Guarrera L, Re Cecconi AD et al; Molecular Genetics Group. Increased ectodysplasin-A2-receptor EDA2R is a ubiquitous hallmark of aging and mediates parainflammatory responses. Nat Commun 2025;16:1898. 10.1038/s41467-025-56918-3 39988718 PMC11847917

[vbag026-B4] Benjamini Y , Hochberg Y. Controlling the false discovery rate: a practical and powerful approach to multiple testing. J R Stat Soc Ser B Methodol 1995;57:289–300. 10.1111/j.2517-6161.1995.tb02031.x

[vbag026-B5] Coupé P , Manjón JV, Lanuza E et al Lifespan changes of the human brain in Alzheimer’s disease. Sci Rep 2019;9:3998. 10.1038/s41598-019-39809-8 30850617 PMC6408544

[vbag026-B6] Dönertaş HM , İzgi H, Kamacıoğlu A et al Gene expression reversal toward pre-adult levels in the aging human brain and age-related loss of cellular identity. Sci Rep 2017;7:5894. 10.1038/s41598-017-05927-4 28724976 PMC5517654

[vbag026-B7] Glass D , Viñuela A, Davies MN et al; MuTHER Consortium. Gene expression changes with age in skin, adipose tissue, blood and brain. Genome Biol 2013;14:R75. 10.1186/gb-2013-14-7-r75 23889843 PMC4054017

[vbag026-B8] GTEx Consortium. Genetic effects on gene expression across human tissues. Nature 2017;550:204–13. 10.1038/nature24277 29022597 PMC5776756

[vbag026-B9] Guzzi PH , Lomoio U, Veltri P. GTExVisualizer: a web platform for supporting ageing studies. Alkan C (ed.). Bioinformatics 2023;39:btad303. 10.1093/bioinformatics/btad303 37154702 PMC10196670

[vbag026-B10] Harris SE , Riggio V, Evenden L et al Age-related gene expression changes, and transcriptome wide association study of physical and cognitive aging traits, in the Lothian birth cohort 1936. Aging (Albany NY) 2017;9:2489–503. 10.18632/aging.101333 29207374 PMC5764388

[vbag026-B11] Jia K , Cui C, Gao Y et al An analysis of aging-related genes derived from the Genotype-Tissue expression project (GTEx). Cell Death Discov 2018;4:26. 10.1038/s41420-018-0093-y PMC6102484 30155276

[vbag026-B12] Korotkevich G , Sukhov V, Budin N et al Fast gene set enrichment analysis. *bioRxiv* 2021, 060012, preprint: not peer reviewed.

[vbag026-B13] Loerch PM , Lu T, Dakin KA et al Evolution of the aging brain transcriptome and synaptic regulation. PLoS One 2008;3:e3329. 10.1371/journal.pone.0003329 18830410 PMC2553198

[vbag026-B14] López-Otín C , Blasco MA, Partridge L et al The hallmarks of aging. Cell 2013;153:1194–217. 10.1016/j.cell.2013.05.039 23746838 PMC3836174

[vbag026-B15] Martinez-Jimenez CP , Eling N, Chen H-C et al Aging increases cell-to-cell transcriptional variability upon immune stimulation. Science 2017;355:1433–6. 10.1126/science.aah4115 28360329 PMC5405862

[vbag026-B16] Nelson PT , Abner EL, Patel E et al The amygdala as a locus of pathologic misfolding in neurodegenerative diseases. J Neuropathol Exp Neurol 2018;77:2–20. 10.1093/jnen/nlx099 29186501 PMC5901077

[vbag026-B17] Poulin SP , Dautoff R, Morris JC et al; Alzheimer’s Disease Neuroimaging Initiative. Amygdala atrophy is prominent in early Alzheimer’s disease and relates to symptom severity. Psychiatry Res 2011;194:7–13. 10.1016/j.pscychresns.2011.06.014 21920712 PMC3185127

[vbag026-B18] Santoro A , Bientinesi E, Monti D. Immunosenescence and inflammaging in the aging process: age-related diseases or longevity? Ageing Res Rev 2021;71:101422. 10.1016/j.arr.2021.101422 34391943

[vbag026-B19] Sims AA , Gurkar AU. DNA damage-induced stalling of transcription drives aging through gene expression imbalance. DNA Repair (Amst) 2023;125:103483. 10.1016/j.dnarep.2023.103483 36921370

[vbag026-B20] Somasundaram I , Jain SM, Blot-Chabaud M et al Mitochondrial dysfunction and its association with age-related disorders. Front Physiol 2024;15:1384966. 10.3389/fphys.2024.1384966 39015222 PMC11250148

[vbag026-B21] Srivastava S. The mitochondrial basis of aging and age-related disorders. Genes (Basel) 2017;8:398. 10.3390/genes8120398 29257072 PMC5748716

[vbag026-B22] Stanfill AG , Cao X. Enhancing research through the use of the Genotype-Tissue expression (GTEx) database. Biol Res Nurs 2021;23:533–40. 10.1177/1099800421994186 33596660 PMC8191158

[vbag026-B23] Stein KC , Morales-Polanco F, Van Der Lienden J et al Ageing exacerbates ribosome pausing to disrupt cotranslational proteostasis. Nature 2022;601:637–42. 10.1038/s41586-021-04295-4 35046576 PMC8918044

[vbag026-B24] Stouffer KM , Grande X, Düzel E et al Amidst an amygdala renaissance in Alzheimer’s disease. Brain 2024;147:816–29. 10.1093/brain/awad411 38109776 PMC10907090

[vbag026-B25] Subramanian A , Tamayo P, Mootha VK et al Gene set enrichment analysis: a knowledge-based approach for interpreting genome-wide expression profiles. Proc Natl Acad Sci USA 2005;102:15545–50. 10.1073/pnas.0506580102 16199517 PMC1239896

[vbag026-B26] Tracy E , Rowe G, LeBlanc AJ. Cardiac tissue remodeling in healthy aging: the road to pathology. Am J Physiol Cell Physiol 2020;319:C166–82. 10.1152/ajpcell.00021.2020 32432929 PMC7468888

[vbag026-B27] Viñuela A , Brown AA, Buil A et al Age-dependent changes in mean and variance of gene expression across tissues in a twin cohort. Hum Mol Genet 2018;27:732–41. 10.1093/hmg/ddx424 29228364 PMC5886097

[vbag026-B28] Yamamoto R , Chung R, Vazquez JM et al Tissue-specific impacts of aging and genetics on gene expression patterns in humans. Nat Commun 2022;13:5803. 10.1038/s41467-022-33509-0 36192477 PMC9530233

[vbag026-B29] Yang J , Huang T, Petralia F et al; GTEx Consortium. Synchronized age-related gene expression changes across multiple tissues in human and the link to complex diseases. Sci Rep 2015;5:15145. 10.1038/srep15145 26477495 PMC4609956

