# Supplementary material for: Age effect explorer: a Shiny application to browse and visualize tissue-specific age-related gene expression changes
Source: Bioinform Adv. 2026 Jan 29;6(1):vbag026. doi: 10.1093/bioadv/vbag026 (PMC12889165; doi:10.1093/bioadv/vbag026)
Supplement: vbag026_Supplementary_Data [file vbag026_supplementary_data.zip › Web service tutorial.pdf]

Shiny dashboard

MENGHUI CHEN

July 2025

1 Introduction

2 Methods

Raw TPM matrices and sample metadata (age, sex, plus available technical/4biological covariates) were downloaded for each tissue from GTEx v10. After reshaping to gene–sample format and applying a $\log_2(\mathrm{TPM}+1)$ transform, we fitted gene-wise linear models by ordinary least squares:

$$y_{ij} = \beta_{0,i} + \beta_{\mathrm{age},i}\,\mathrm{Age}_j + \beta_{\mathrm{sex},i}\,\mathrm{Sex}_j + \sum_k \gamma_{k,i}\,C_{kj} + \varepsilon_{ij},$$

Here, y_{ij} is the transformed TPM for gene i in donor j, $\mathrm{Sex}_j \in \{0,1\}$ is a binary variable with 1 for male and 0 for female donors, and C_{kj} denotes additional covariates. Genes with negligible variance or with more than 90% zero values were excluded. P-values for $\beta_{\mathrm{age},i}$ and $\beta_{\mathrm{sex},i}$ were adjusted using the Benjamini–Hochberg procedure, and associations with FDR < 0.05 were considered significant.

3 Implementation

GTEx Explorer is built in R/Shiny with `plotly` and `DT` for interactive plots and tables. Precomputed tables of regression coefficients and FDRs drive the interface. The app runs locally in RStudio and is hosted at `https://menghui.shinyapps.io/shinyProject/`.

4 Results and Use Case

The GTEx Explorer tool facilitates expeditious investigation of gene expression changes associated with age and sex in 54 human tissues. To illustrate its functionality, we examined two examples from the GTEx v10 dataset.

To validate the established biological finding, we performed an examination of the expression of EDA2R (EDA, Ectodysplasin A) in whole blood, where the analysis by [1] confirmed its correlation with age and demonstrated a positive

association between the expression of EDA2R in blood. Consistently, our GTEx Explorer analysis of whole blood identified EDA among the top positively age-correlated genes (Age FDR = 3.86×10^{-6}) within a set of 98 significantly age-associated genes. For a potential novel finding, R-spondin 4 (RSPO4) was investigated, which is significantly age-associated in normal breast mammary tissue (Age FDR = 2.60×10^{-5}) within a total of 224 age-associated genes in our GTEx Explorer analysis. [2] demonstrated that RSPOs stimulate the canonical Wnt/β-catenin pathway in disease development. In breast cancer, RSPO4 is overexpressed in triple-negative and metaplastic tumors, where it promotes tumor growth [3]. Therefore, age-related up-regulation of RSPO4 in normal breast mammary tissue may enhance Wnt signaling and contribute to the increased risk of breast cancer in elderly women.

Next, we performed the GO enrichment analysis of age-associated genes. First, the GO analysis result of 98 significantly age-associated genes in whole blood FDR < 0.05 showed that only those decreasing with age were significant. All enriched terms are related to T cell processes: T-cell receptor signaling, activation and regulation of immune response, lymphocyte costimulation, etc. This points to immunosenescence (aging of the immune system): Lower expression of T-cell gene may reduce receptor diversity and weaken immune responses in older adults, contributing to diseases such as Alzheimer's. Secondly, GO analysis of 7,632 significantly age-associated genes in the brain amygdala FDR < 0.05 was conducted. Genes that decrease significantly with age were enriched for core maintenance functions: protein degradation, histone modification, RNA processing and splicing, mitochondrial gene expression, and cell division, indicating a loss of cellular maintenance. In contrast, genes that increase significantly with age were assigned to immune defense and cell killing functions, highlighting a rise in neuroinflammatory activity. Together, these changes suggest that aging in the brain amygdala involves weak repair systems and increased immune activity, a combination that may be the basis for neurodegenerative risk.

In general, these examples demonstrate the ability of GTEx Explorer to summarize existing findings and identify new age- or gender-related gene expression changes, providing researchers with valuable tools to effectively navigate the complexity of large-scale transcriptomic data.

5 Availability and Requirements

User Interface Overview

The GTEx Explorer user interface is divided mainly into two sections: *Per-Tissue Summary* and *Gene Analysis*, where focuses are different: the former pays attention to the overview of differential expression at the overall tissue level, while the latter can delve into the performance of individual genes in a single tissue and across tissues.

In the "Per tissue summary" tab, users choose "Age" or "Sex" at the top, which immediately updates a table summarizing the differential expression of

each tissue, and we only consider significant genes with FDR < 0.05. For "Age", genes with positive regression coefficients are counted as up-regulated, and those with negative coefficients are counted as down-regulated. Meanwhile, for "Sex", genes with positive coefficients indicate male-biased expression and those with negative coefficients indicate female-biased expression. This table shows each tissue's name, sample size, number of significant genes in the chosen direction, total significant genes, and percentage of up-regulated genes. Also, the percentage column has a colored bar to show the data visually. Below the table is an interactive horizontal bar chart, where it sorts all tissues by the up-regulated gene proportion ratio, and the bar colors are distinguished according to the summary dimension (age or gender). The mouse can move around to display the precise values. Users can click the "Download" button to export the current summary table in CSV format. The file name will automatically include the tissue name, summary type, and date information. Through this module, researchers can quickly obtain the full picture of the expression of any tissue in the dimensions of age or sex without programming, which provides guidance for analysis.

The "Gene Analysis" TAB is based on the gene level, which enables users to view the expression dynamics of one gene in the specified tissue and compare its age/gender effects across tissues. The tissue can still be selected via the drop-down menu. The "Show age summary" and "Show sex summary" checkboxes below it can display the brief statistics of significant genes within the tissue (the same as the summary view in the previous module, but only for the current tissue). Then, the Gene-level P-value table for the selected tissue can be generated, while users can also select the sorting by age FDR or gender FDR through the radio button "Rank genes by". After that, the system will display the regression results of all genes in the corresponding tissue, which includes Age p-value, Age FDR, Age effect, Gender p-value, Gender FDR and Gender Effect, where the "Age Effect" indicates whether gene expression increases ("positive") or decreases ("negative") with age, and "Gender Effect" specifies the sex with higher gene expression ("male" if expression is higher in males, "female" if higher in females). For p-values and FDR < 0.1, the table will automatically retain two significant figures using scientific notation, while the remaining values will display two decimal places. Users can click the "Download" button above the table to export the entire table as a CSV file. The file name will indicate the organization name and the sorting method.

After loading the gene table, if the user selects a certain gene, the interface will present three switchable visual plots: expression and age scatter plot, age-specific box plot, and residual plot. For the scatter plot, each sample point shows the relationship between the $\log_2(\mathrm{TPM}+1)$ expression value of the gene and age, and is plotted in different colors according to sex. The system superimposes two parallel regression lines (corresponding to males and females respectively) on the plot, while they are directly drawn using the precalculated estimated intercept, estimated age coefficient, and gender coefficient. If switched to the age-group box plot, the system will group the samples into age intervals such as "0–19", "20–29", "30–39", and so one, with the last interval

labeled as “70+”. The box plot displays the log-transformed expression distribution of males and females in each group, respectively, to observe the median, distribution differences, and outliers among different age groups. In addition, the residual plot shows how the difference between the observed expression of each sample and the predicted expression of the model varies with age. If the checkbox “Include sex effect” is selected, the model takes into account sex when computing predicted values; otherwise, sex is omitted from the regression. The titles of all plots are generated as “`<Gene Name> in <Tissue Name>`”, and the subheadings display FDRs, their rankings and percentiles of the age and gender effects of the gene in the tissue. Below the graphic, export options are provided in formats that include PNG, PDF, SVG, and JPG.

Below the visualization graph, the system also presents a cross-tissue summary table of one specific gene. This table lists the Age Effect Coefficient, Age FDR, Sex Effect Coefficient and Sex FDR of this gene in all tissues. By default, they are sorted in ascending order of age FDR, which is convenient to quickly identify which tissue has the most significant age association of this gene. Users can also click on any column title to reorder the table. Users can also click the ”Download” button to export the results as a CSV file for subsequent analysis.

If the user has not yet chosen a specific gene, they can first see which genes in a tissue are most related to age or gender by using the “Rank genes by” option and clicking “Generate gene-level p-value table”. The genes ranked by FDR will appear in a table. Users can easily pick an interesting gene from the top results and generate scatter plots, box plots, residual plots, and cross-tissue summaries.

Overall, the “Per-Tissue Summary” module gives a general overview of gene expression differences across tissues, while the “Gene Analysis” module helps users look deeper into how a single gene behaves in one tissue or across different tissues.

References

[1] M. C. Barbera, L. Guarrera, A. D. Re Cecconi, and et al. Increased ectodysplasin-a2-receptor eda2r is a ubiquitous hallmark of aging and mediates parainflammatory responses. *Nature Communications*, 16:1898, 2025. doi: 10.1038/s41467-025-56918-3. URL `https://doi.org/10.1038/s41467-025-56918-3`.

[2] Z. He, J. Zhang, J. Ma, L. Zhao, X. Jin, and H. Li. R-spondin family biology and emerging linkages to cancer. *Annals of Medicine*, 55(1):428–446, December 2023. doi: 10.1080/07853890.2023.2166981.

[3] J. M. Tocci, C. M. Felcher, M. E. García Solá, and E. C. Kordon. R-spondin-mediated wnt signaling potentiation in mammary and breast cancer development. *IUBMB Life*, 72(8):1546–1559, August 2020. doi: 10.1002/iub.2278. Epub 2020 Mar 31.
