# Supplementary material for: Age effect explorer: a Shiny application to browse and visualize tissue-specific age-related gene expression changes
Source: Bioinform Adv. 2026 Jan 29;6(1):vbag026. doi: 10.1093/bioadv/vbag026 (PMC12889165; doi:10.1093/bioadv/vbag026)
Supplement: vbag026_Supplementary_Data [file vbag026_supplementary_data.zip › Supplementary figures.pdf]

Supplementary figures for:

Age Effect Explorer: A Shiny application to browse and visualize tissue-specific age-related gene expression changes

Menghui Chen[1,#], Mingrui Li[1,#], Ronnie Y. Li[2], Jie Jiang[3], Zhaohui S. Qin[1,*]

[1] Department of Biostatistics and Bioinformatics, Emory University, Atlanta, GA 30322
[2] Stravitz-Sanyal Institute for Liver Disease and Metabolic Health, Virginia Commonwealth University, Richmond, VA 23298
[3] Department of Cell Biology, Emory University, Atlanta, GA 30322
# These authors contributed equally to this work.
* Corresponding author.

Contents:

Supplementary Figure 1. Age distributions of GTEx donors.

Supplementary Figure 2. Gene set enrichment analysis of cytoplasmic ribosomal proteins.

Supplementary Figure 3. Summary of differentially expressed aging genes (DEGs) across tissues.

Supplementary Figure 4. Gene set enrichment analysis (GSEA) in amygdala and other brain tissues.

Supplementary Figure 5. Sensitivity analyses (power analysis and permutation tests).

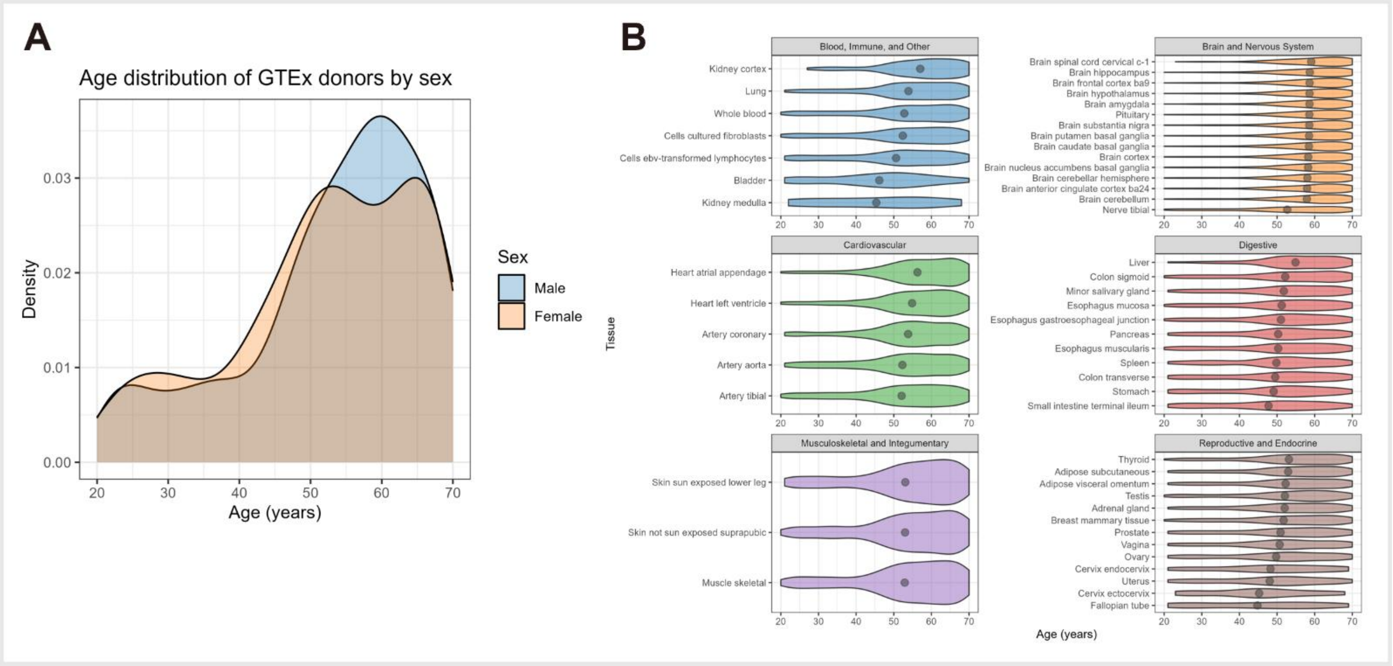

Supplementary Figure 1. Age distributions of GTEx donors. **A.** Density plot of age distributions of GTEx donors by biological sex. **B.** Individual, tissue-specific age distributions of GTEx donors for all 54 GTEx tissues. Tissues are broadly grouped into six categories based on anatomical location and primary function. Gray dots indicate mean age for each tissue.

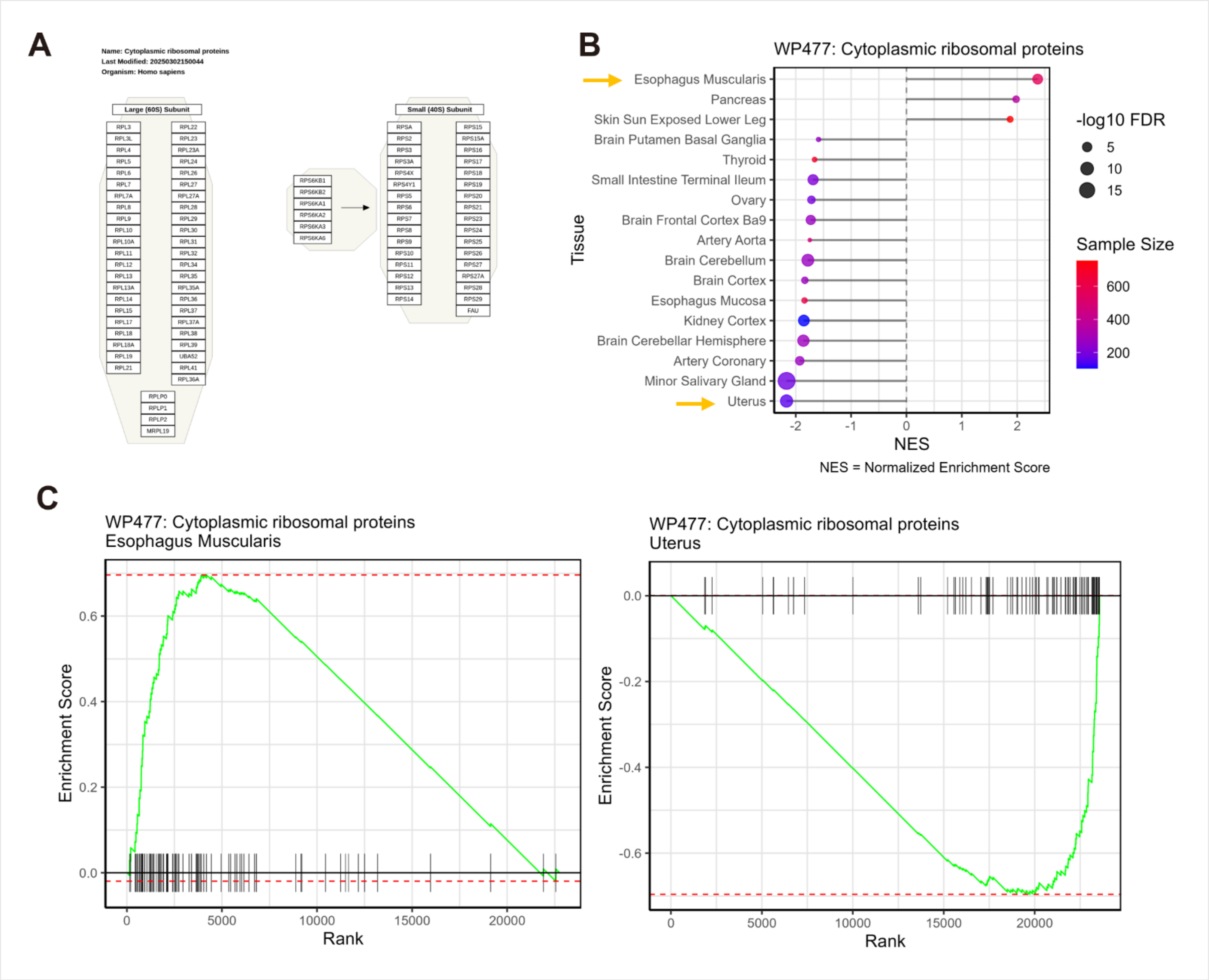

Supplementary Figure 2. Gene set enrichment analysis of cytoplasmic ribosomal proteins (WP477). **A.** Visualization of the genes within this pathway. **B.** The 17/50 tissues for which this pathway is statistically significant (padj < 0.05), including 14 with negative NES and 3 with positive NES. Normalized enrichment scores are shown on the x-axis. Sample size represents the number of donors available for each tissue. **C.** Individual enrichment score plots of this pathway in the most upregulated (esophagus muscularis) and downregulated (uterus) tissues.

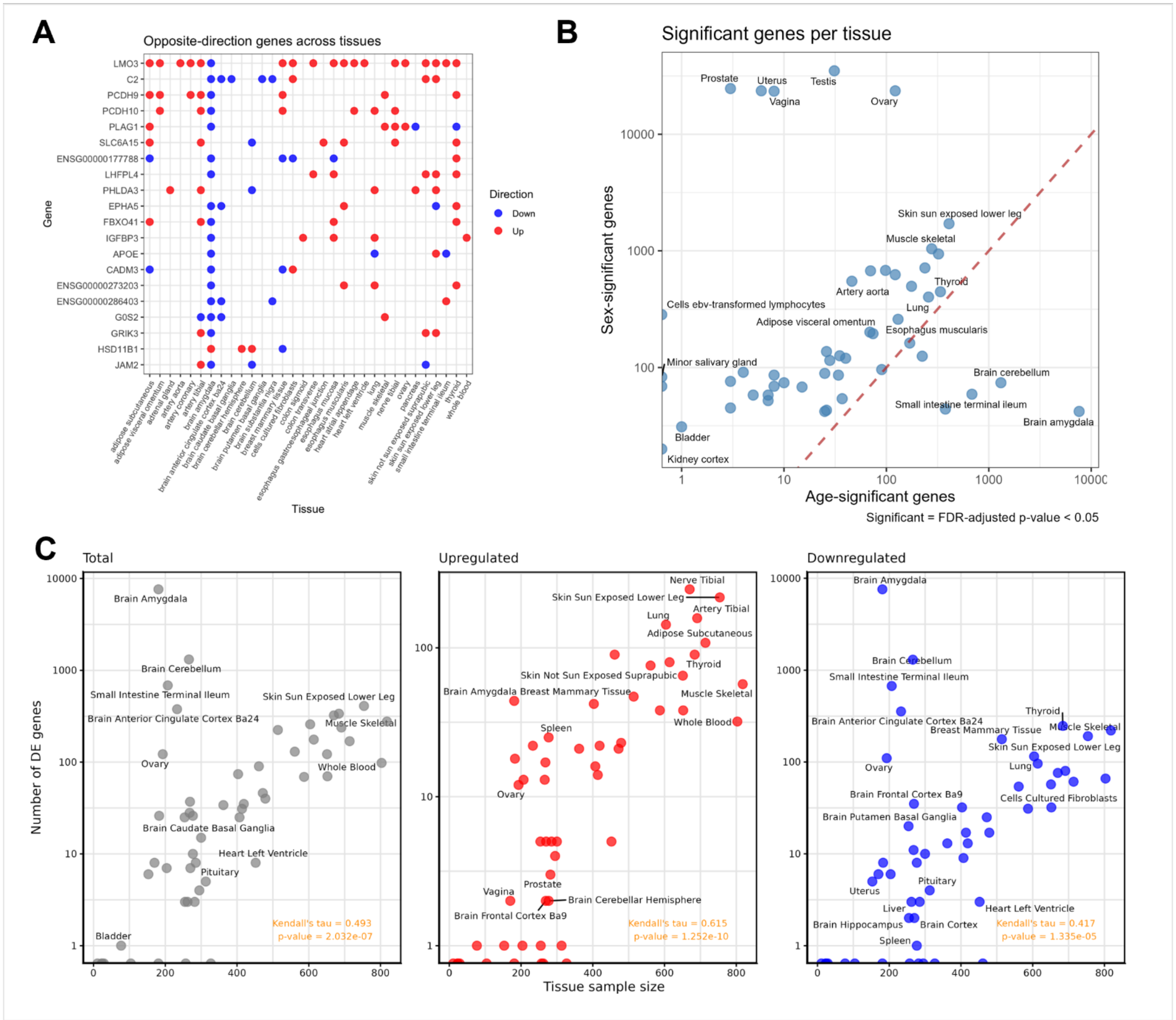

Supplementary Figure 3. Summary of differentially expressed aging genes (DEGs) across tissues. **A.** Examples of genes that show an opposite age effect in at least two GTEx tissues. **B.** Relationship between the number of age-significant (p-adj < 0.05) genes and number of sex-significant (p-adj < 0.05) genes in each tissue. **C.** Relationship between the tissue sample size and total number of age-significant DEGs (left), age-upregulated DEGs (middle), and age-downregulated DEGs (right).

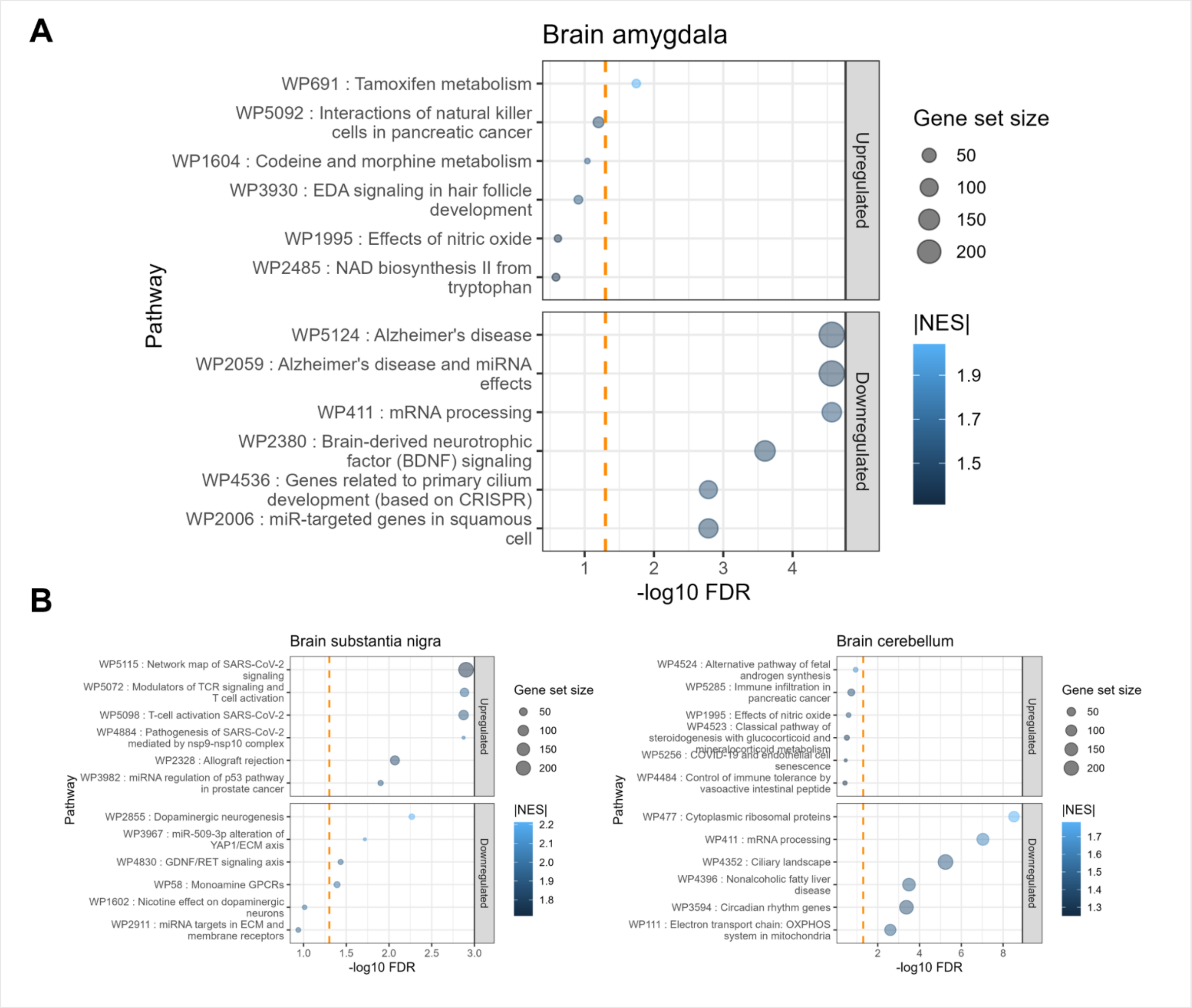

Supplementary Figure 4. A. Gene set enrichment analysis (GSEA) of aging genes in the amygdala reveals enrichment for Alzheimer's disease-related terms among downregulated genes. This finding is supported by literature evidence showing the amygdala's significant involvement in Alzheimer's disease and neurodegeneration. **B.** GSEA on aging genes in other brain tissues did not yield the same enrichment for Alzheimer's disease, suggesting that the amygdala's result is unique and biologically meaningful.

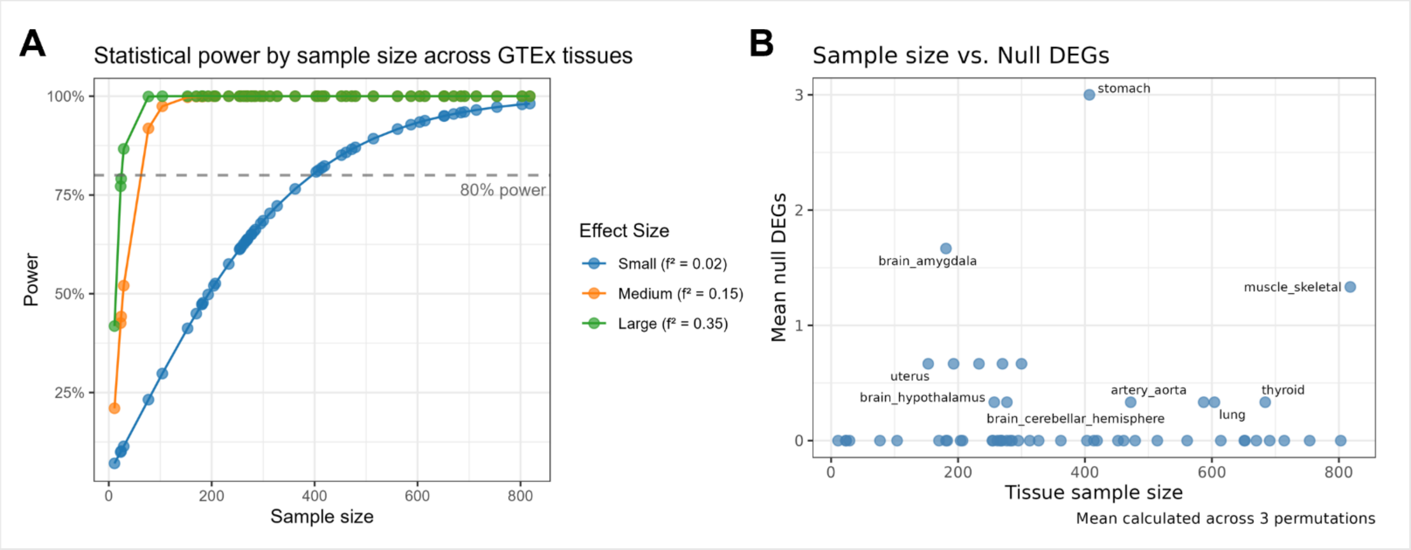

Supplementary Figure 5. A. Power analysis using Cohen's standard effect sizes for linear regression and one predictor (age or sex) shows that statistical power increases steeply with sample size, particularly for medium and large effects. **B.** Permutation testing with shuffled age labels corroborates true age effect signal over technical artifacts, as all tissues have extremely low numbers of "null" DEGs under the permuted ages.
